# An insertion variant of *MGMT* disrupts a STAT1 binding site and confers susceptibility to glioma

**DOI:** 10.1186/s12935-021-02211-4

**Published:** 2021-09-20

**Authors:** Liming Huang, Wenshen Xu, Danfang Yan, Xi Shi, Xin You, Jiaqi Xu, Pingping You, Yuanyuan Ke, Lian Dai

**Affiliations:** 1 Department of Medical Oncology, The First Affiliated Hospital of Fujian Medical University, #20 Chazhong Road, Fuzhou, 350005 China; 2 Molecular Oncology Research Institute, The First Affiliated Hospital, Fujian Medical University, Fuzhou, 350005 China; 3 Department of Laboratory Medicine, The First Affiliated Hospital, Fujian Medical University, Fuzhou, 350005 China; 4 Department of Radiation Oncology, The First Affiliated Hospital, College of Medicine, Zhejiang University, Hangzhou, 310003 China; 5 Department of Medicine, The Third Affiliated People’s Hospital, Fujian University of Traditional Chinese Medicine, #363 Guobin Road, Fuzhou, 350108 China

**Keywords:** Glioma, *MGMT*, Expression quantitative trait locus, *STAT1*, Susceptibility

## Abstract

**Background:**

*O*^6^-methylguanine-DNA methyltransferase (MGMT) is a pivotal enzyme for repairing DNA alkylation damage. Epigenetic modification of *MGMT* has been well known as a promising prognostic biomarker for glioma. However, the significance of genetic variations of *MGMT* in glioma carcinogenesis has not been fully elucidated.

**Methods:**

The associations between expression quantitative trait loci (eQTLs) of *MGMT* and glioma susceptibility were evaluated in a case–control study of 1056 individuals. The function of susceptibility locus for glioma was explored with a set of biochemical assays, including luciferase reporter gene, EMSA and supershift EMSA, ChIP, and siRNA knockdown.

**Results:**

We found that rs11016798 TT genotype was associated with a significantly decreased risk of glioma (OR = 0.57, 95% CI 0.39–0.85; *P* = 0.006). Stratification analyses indicated that the association between rs11016798 and glioma was more pronounced in males (OR = 0.62, 95% CI 0.40–0.97; *P* = 0.035), older subjects (OR = 0.46, 95% CI 0.27–0.80; *P* = 0.006), WHO grade IV glioma (OR = 0.58, 95% CI 0.35–0.96; *P* = 0.033), and IDH wildtype glioma (OR = 0.43, 95% CI 0.21–0.88; *P* = 0.022). We characterized an insertion variant rs10659396 in the upstream of *MGMT* as a causative variant. The risk allele rs10659396 ins allele was demonstrated to downregulate *MGMT* expression by disrupting a STAT1 binding site. Knockdown of *STAT1* remarkably attenuated *MGMT* expression. Moreover, the rs10659396 allele-specific positive correlation was observed between the expression of *STAT1* and *MGMT* in population.

**Conclusions:**

The study demonstrates that an insertion variant of *MGMT* rs10659396 confers susceptibility to glioma by downregulating MGMT expression through disrupting a STAT1 binding site.

**Graphic abstract:**

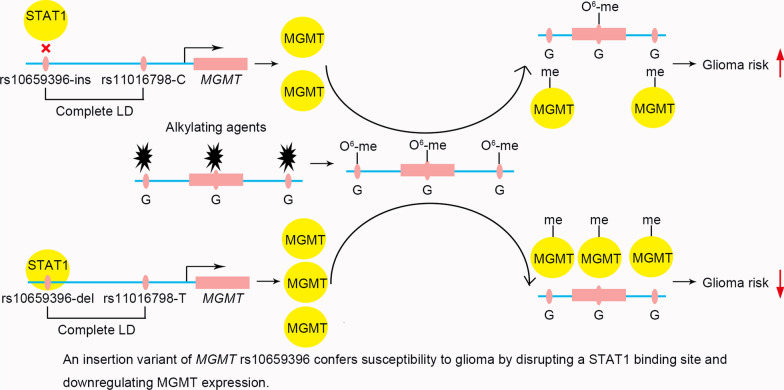

**Supplementary Information:**

The online version contains supplementary material available at 10.1186/s12935-021-02211-4.

## Background

Glioma is the most frequent primary tumour of the central nervous system [1]. Because of anatomical particularity and highly aggressive biological behavior, glioma is considered to be one of the most devastating cancers. Despite the substantial progress made in research on glioma in the past decades, the prognosis of most glioma patients remains dismal [1, 2]. Therefore, it is an urgent need to further reveal the mechanism of glioma carcinogenesis.

Accurate DNA repair is essential for the maintenance of genome integrity. Defects in DNA repair pathway induced by genetic or epigenetic alterations have been reported to be involved in carcinogenesis of several kinds of cancer [3]. Alkylating agents are a class of potent carcinogens that exist widely in the internal and external environments. Although at low levels, much of the population of the world is exposed to alkylating agents continuously. The principal mutagenic damage induced by alkylating agents is *O*^6^-alkylguanine in DNA [4]. *O*^6^-methylguanine-DNA methyltransferase (MGMT) is a critical DNA repair enzyme which protects cells against the carcinogenic effects of alkylating agents. It repairs DNA alkylation damage by transferring the methyl moiety at *O*^6^ site of guanine onto its cysteine residues irreversibly. Upon this process, MGMT protein is consumed [5]. Due to this suicide mechanism, the sufficient supply of MGMT is required for the efficient repair of DNA alkylation damage. Consistently, decreased expression of *MGMT* was observed in several kinds of cancer [6]. In addition, it was found that overexpression of *MGMT* can significantly reduce hepatic cancer development [7]. On the other hand, in glioma patients undergoing the alkylating agent temozolomide treatment, low MGMT expression was found to improve patient survival and treatment response, which may be due to the incomplete repair of temozolomide induced DNA alkylation damage in glioma cells [8].

Epigenetic modification is the most common determinant of inactivation of *MGMT* in glioma [6]. Promoter methylation of *MGMT* has been well known as a valuable prognostic factor and predictor of response to temozolomide for glioma [8]. Nevertheless, discordance between *MGMT* promoter methylation status and gene expression was observed in numerous studies [8]. It indicates that alternative mechanisms for *MGMT* expression regulation, such as genetic or post-transcriptional modulation, should merit further research. For instance, common loss of one allele of chromosome 10q26 on which the *MGMT* gene resides has been reported in primary glioblastoma [9]. Moreover, several genetic variations of *MGMT* were revealed to affect gene expression [10, 11]. On the other hand, several studies have demonstrated that genetic variations affecting gene expression, known as expression quantitative trait loci (eQTLs), are more likely to be traits-associated factors [12, 13]. Therefore, it is worthwhile to evaluate the role of the eQTLs of *MGMT* in glioma development.

In this study, we investigated whether the eQTLs of *MGMT* identified from brain tissues confer susceptibility to glioma. We discovered an eQTL of *MGMT* rs11016798 that was associated with glioma susceptibility. Using stratification analyses, we observed more pronounced decreased risk of glioma in males, subjects older than 50-year, WHO grade IV glioma, and IDH wildtype glioma subgroups. In addition, we characterized an insertion variant rs10659396 in the upstream of *MGMT* as a causative variant because it disrupted a STAT1 binding site, resulting in downregulation of *MGMT* expression.

## Methods

### Study subjects

This study included 402 glioma patients and 654 cancer-free population controls, all of whom were unrelated Southern Han Chinese (CHS). Part of this case–control panel was reported in our previous molecular epidemiologic study on glioma [14]. We recruited patients who were histopathologically confirmed glioma from January 2010 to July 2016 at the First Affiliated Hospital, Fujian Medical University and the First Affiliated Hospital, College of Medicine, Zhejiang University. At initial diagnosis, at least two local pathologists confirmed the pathologic diagnosis for each patient according to the 2007 WHO classification of tumours of the central nervous system [15]. The 2016 WHO classification of tumours of the central nervous system was applied to reclassify the pathologic diagnosis for each patient in this study [16]. Controls were cancer-free individuals recruited in the same region during the period of patients recruitment. Baseline clinical characteristics of patients and controls are summarized in Additional file 1. Written informed consent was obtained from all subjects and this study was approved by the Institutional Review Board of the First Affiliated Hospital, Fujian Medical University.

### Selection of candidate genetic variations

The eQTL Browser of BrainSeq project (http://eqtl.brainseq.org/), which identified eQTLs in 412 brain tissues using RNA sequencing and genotype data, was used to choose brain eQTLs of *MGMT* as candidates [17]. The “DLPFC-Control” database consisted of eQTLs identified in 237 control brain samples was selected for analysis. We used gene symbol “*MGMT*” as search term, and applied a *P* value cutoff of 0.05 after Bonferroni correction. Then, we picked out the common eQTLs with minor allelic frequency (MAF) > 10% in CHS population to assess the linkage disequilibrium (LD) status using Haploview v4.2 software [18]. Tag-eQTLs were selected for genotyping.

### Genotype analysis

A commercial Tiangen TIANamp Genomic DNA kit (Tiangen Biotech., Beijing, China) was used to isolate genomic DNA from peripheral blood lymphocytes. Genotypes of the tag-eQTLs were determined by Sequenom MassARRAY iPLEX platform (Sequenom Inc., San Diego, CA, USA). For genotyping quality control, we implemented several measures. DNA samples of the cases and controls were mixed in every assay plate and blinded for disease status. Positive and negative (no DNA) controls were included in every assay plate. A 5% masked random sample was repeatedly tested, and the reproducibility was higher than 99%.

### In silico analysis

Several tracks of the UCSC Genome Browser (http://www.genome.ucsc.edu/) were applied to explore potential causative variant [19]. The track, DNaseI Hypersensitive Site Master List (125 cell types) from ENCODE/Analysis, shows DNaseI hypersensitive sites which are markers of regulatory DNA elements [20]. The ORegAnno track displays potential regulatory regions [21]. The UCSF Brain DNA Methylation track depicts the H3K4me3 and DNA methylation signal [22]. JASPAR database (http://jaspar.genereg.net/) was exploited to predict the transcription factor binding to the interest region [23]. The sequences for analysis were rs11016798 5ʹ-AAG AAC CCA (C/T) TGT ACT TTG-3ʹ and rs10659396 5ʹ-TCA CTC CTC (-/CTT) TAA CCC ACT-3ʹ. To examine whether the potential causative variant rs10659396 has a functional effect on *MGMT* expression, we extracted the gene expression data of *MGMT* and genotype data of rs10659396 from the “DLPFC-Control” database of BrainSeq project [17].

### Luciferase reporter gene assays

For luciferase reporter gene assays, we subcloned a 249-bp DNA fragment, which contains rs10659396 ins allele and corresponds to a potential regulatory region OREG1737695 predicted by the ORegAnno track, into the pGL3-promoter vector (Promega, Madison, WI, USA). The resultant construct was designated as P-ins. Then, we created the P-del construct containing rs10659396 del allele by means of site-directed mutagenesis. The construct P-del, P-ins, and an empty pGL3-promoter vector was respectively cotransfected with pRL-TK vector (Promega) into 293T cells with Lipofectamine reagent (Life Technologies, Carlsbad, CA, USA). Three independent transfection experiments were done for each construct, and each was carried out in triplicate. The luciferase activity was analyzed by a Dual-Luciferase Reporter Assay System (Promega). The 293T cells, which were obtained from X-Y Biotechnology Corporation (Shanghai, China), were authenticated by DNA finger printing analysis and tested for mycoplasma contamination.

### Electrophoretic mobility-shift assays

The double-stranded and 5ʹ biotin-labeled oligonucleotides corresponding to rs10659396 del (Probe-del) or ins (Probe-ins) sequences were synthetized and incubated with U251 cell nuclear extract using a BersinBio Electrophoretic Mobility-Shift Assays (EMSA) Kit (BersinBio Biotech., Guangzhou, Guangdong, China). For competition assays, non-labeled oligonucleotides at 200-fold molar excess were added to the reaction before the biotin-labeled probes. The antibody against STAT1 (Abcam, Cambridge, UK) was used for supershift assays. The reaction mixture was separated on 5% PAGE, and detected by Streptavidin-HRP Conjugate (BersinBio Biotech.). The oligonucleotide probes are shown in Additional file 2. The U251 cells, which were obtained from X-Y Biotechnology Corporation (Shanghai, China), were authenticated by DNA finger printing analysis and tested for mycoplasma contamination.

### Chromatin immunoprecipitation assays

U251 cells carrying rs10659396 del/del genotype were fixed with 1% formaldehyde. Then, cell lysates were sonicated and subjected to immunoprecipitation using a BersinBio Chromatin Immunoprecipitation (ChIP) Kit (BersinBio Biotech.) and antibodies against STAT1 or nonspecific rabbit IgG (Abcam). Purified DNA was analyzed by PCR. We also examined whether STAT1 binds to the randomly selected region of β-actin promoter by PCR to validate the nonrandom binding of STAT1 to rs10659396. The PCR primers are shown in Additional file 2.

### RNA interference and gene expression analysis

Three silencing RNAs (siRNAs) were designed to knockdown *STAT1* expression. The STAT1 siRNAs and negative control siRNA were respectively transfected into U251 cells using Lipofectamine reagent (Life Technologies). After 48 h, the expression levels of *STAT1* and *MGMT* were analyzed by RT-qPCR and Western blotting. For analyses of *STAT1* and *MGMT* mRNA levels, total RNA was isolated by Trizol (Life Technologies) and converted to complementary DNA. Then, mRNA levels were measured in triplicate with TB Green Premix Ex Taq (Takara Bio., Shiga, Japan). For analyses of STAT1 and MGMT protein levels, protein was extracted and separated on SDS-PAGE. PVDF membranes were used to blot the proteins, followed by incubation with primary antibody against STAT1 or MGMT (Abcam). Then, the protein bands were revealed with HRP-conjugated secondary antibody and visualized using enhanced chemiluminescence. GAPDH was applied as a reference. The siRNAs and primers for RT-qPCR are summarized in Additional file 2.

### Correlation analysis between STAT1 and MGMT expression

The Gene Expression Omnibus (GEO, http://www.ncbi.nlm.nih.gov/geo/) was used to extract the normalized gene expression data of *STAT1* and *MGMT* [24]. The GEO accession number is GSE6536 which includes whole-genome gene expression levels measured in 270 unrelated HapMap individuals [25]. The target ID of *MGMT* is GI_4505176-S. The target ID of *STAT1-α* and *STAT1-β* is GI_21536299-I and GI_21536300-I respectively. The gene expression data normalized by a median normalization method across all HapMap individuals was used in this study.

### Statistical analysis

Logistic regression model was used to assess the associations between genotypes and glioma risk using Statistic Analysis System software (version 9.4, SAS Institute, Cary, NC, USA). Bonferroni correction was performed to correct the *P* values for multiple testing. A t-test was used to examine the differences in gene expression level and luciferase reporter gene expression. Pearson correlation was applied for analysis of *STAT1* and *MGMT* expression correlation. All statistical tests were two sided, and *P* value of < 0.05 was used as the criterion of statistical significance.

## Results

We used data from BrainSeq project to select brain eQTLs of *MGMT* for analysis. Four common variants rs11016798, rs487120, rs557311, and rs1711654 identified as tag-eQTLs were genotyped in this study. Associations of the 4 tag-eQTLs with *MGMT* expression levels were shown in Additional file 3. The genotype frequencies of rs11016798 were summarized in Table 1. We found that the genotype frequencies of rs11016798 in cases were 34.61% (CC genotype), 51.54% (CT genotype), and 13.85% (TT genotype), which differed significantly from those in controls [29.02% (CC), 49.61% (CT), and 21.37% (TT), *P* = 0.007]. Notably, multivariate logistic regression analysis showed that subjects with rs11016798 TT genotype had a significantly decreased risk of glioma (OR = 0.57, 95% CI 0.39–0.85; *P* = 0.006) compared with those with rs11016798 CC genotype. In a recessive model, the adjusted OR was 0.61 (95% CI 0.43–0.86; *P* = 0.005). The association for rs11016798 remained significant after Bonferroni correction for four testing. Unfortunately, no evidence was found for the associations between the other 3 tag-eQTLs and glioma susceptibility (Table 1).

**Table 1:** Genotype frequencies of rs11016798, rs487120, rs557311 and rs1711654 and their associations with glioma risk

Variants	Genotype^a^	Patients (n = 402) No. (%)	Controls (n = 654) No. (%)	OR^b^ (95 %CI)	*P*
rs11016798	CC	135 (34.61)	186 (29.02)	1.00 (reference)	
	CT	201 (51.54)	318 (49.61)	0.90 (0.68–1.21)	0.491
	TT	54 (13.85)	137 (21.37)	0.57 (0.39–0.85)	0.006
	TT vs. CC + CT			0.61 (0.43–0.86)	0.005
rs487120	TT	152 (38.00)	265 (40.65)	1.00 (reference)	
	CT	190 (47.50)	294 (45.09)	1.13 (0.86–1.49)	0.385
	CC	58 (14.50)	93 (14.26)	1.05 (0.71–1.56)	0.808
rs557311	GG	167 (41.75)	309 (47.61)	1.00 (reference)	
	AG	185 (46.25)	266 (40.99)	1.28 (0.98–1.68)	0.072
	AA	48 (12.00)	74 (11.40)	1.13 (0.74–1.73)	0.580
rs1711654	TT	190 (47.62)	325 (50.31)	1.00 (reference)	
	CT	169 (42.36)	251 (38.85)	1.17 (0.89–1.53)	0.268
	CC	40 (10.02)	70 (10.84)	1.01 (0.65–1.57)	0.963

*OR* odds ratios,* CI* confidence interval

^a^Some samples failed to genotype

^b^Data were calculated by logistic regression, adjusted for age and sex

**Fig. 1:**
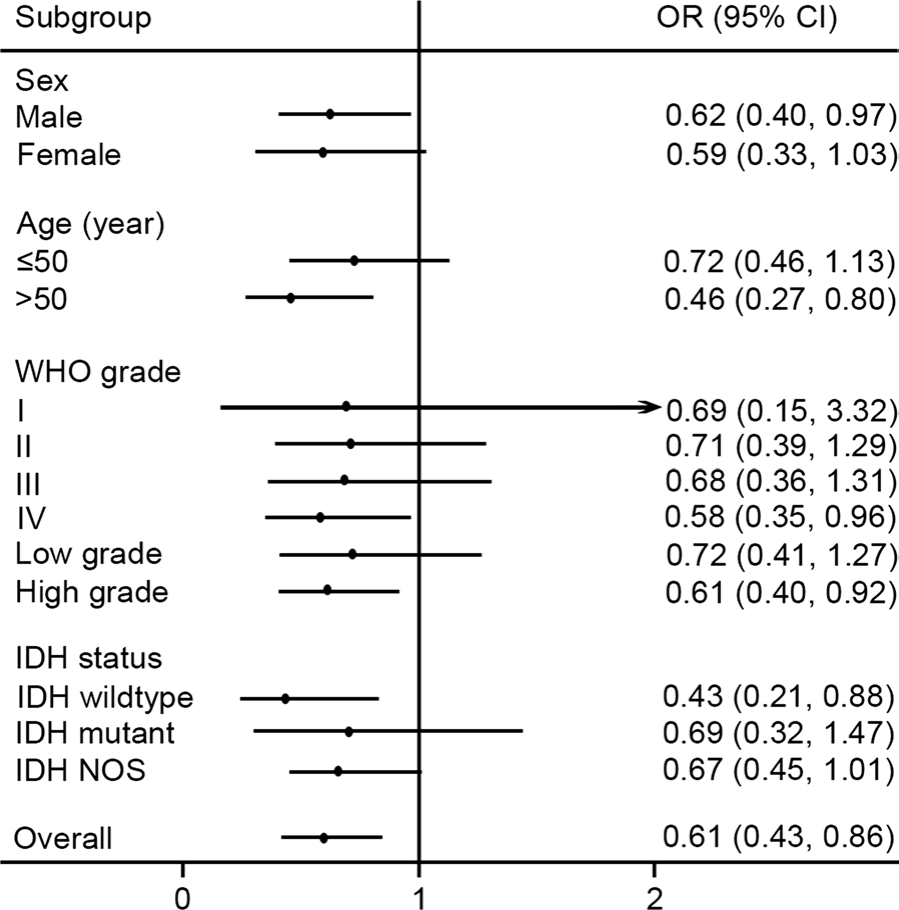
Stratification analyses for rs11016798 based on sex, age, WHO grade, and IDH status in a recessive model. Central black dots represent ORs. Horizontal lines indicate 95% CIs

Then, we performed stratification analyses for rs11016798 in a recessive model. As shown in Fig. 1, we found that subjects with rs11016798 TT genotype were more likely to have decreased risk of glioma in all subgroups. Significantly decreased risk of glioma was observed in males, subjects older than 50-year, WHO grade IV glioma, and IDH wildtype glioma with adjusted OR of 0.62 (95% CI 0.40–0.97; *P* = 0.035), 0.46 (95% CI 0.27–0.80; *P* = 0.006), 0.58 (95% CI 0.35–0.96; *P* = 0.033), and 0.43 (95% CI 0.21–0.88; *P* = 0.022) respectively. When glioma was further categorized as low grade glioma and high grade glioma, a remarkably reduced risk of glioma was just observed in high grade glioma with adjusted OR of 0.61 (95% CI 0.40–0.92; *P* = 0.019). No significant association was found in other subgroups, which might be attributed to the relatively small sample size within a given stratum.

In silico analysis revealed that rs11016798 locates 9 kb upstream of *MGMT*, and there is no DNaseI hypersensitive site at rs11016798 region (Additional file 4). Moreover, neither potential regulatory region nor transcription factor binding site was displayed at rs11016798 region by ORegAnno. Furthermore, deficiency of H3K4me3 also supports the contention that rs11016798 may just be a genetic marker without functional characteristics. In the 15 kb upstream of *MGMT*, we found another variant rs10659396 that is in perfect linkage disequilibrium with rs11016798 (r^2^ = 1.00, Dʹ = 1.00) in multiple populations including Chinese. Corresponding to rs11016798 T allele is rs10659396 del allele. As shown in Additional file 5, rs10659396 locates in a DNaseI hypersensitive region. There are three potential regulatory elements predicted by ORegAnno. Moreover, H3K4me3 enrichment was also displayed in this region. These results implied that rs10659396 is probably the underlying causative variant with functional characteristics.

**Fig. 2:**
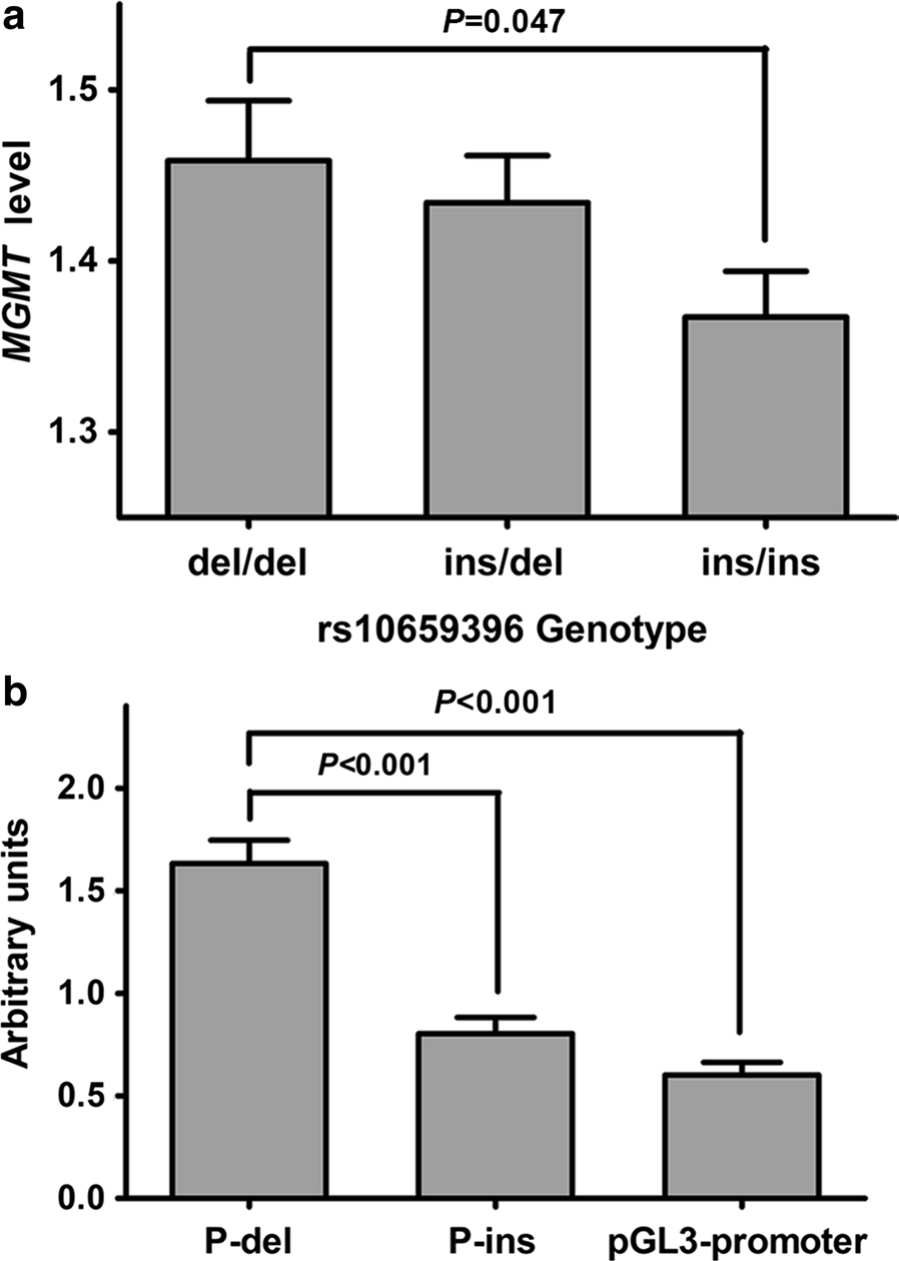
**a** *MGMT* expression levels as a function of rs10659396 genotype. **b** Reporter gene assays with constructs containing the rs10659396 del allele (P-del) or ins allele (P-ins) in 293T cells. Columns indicate means; Bars represent SE

We used the gene expression data of *MGMT* and genotype data of rs10659396 from BrainSeq project to investigate the association between rs10659396 genotypes and *MGMT* expression levels in population. As shown in Fig. 2a, the *MGMT* expression levels of subjects with rs10659396 del/del genotype were significantly higher than those with ins/ins genotype, with the values (mean ± SE) being 1.46 ± 0.04 versus 1.37 ± 0.03 (*P* = 0.047). Meanwhile, luciferase reporter gene assays were performed to investigate whether rs10659396 has an impact on gene expression in cell line (Fig. 2b). Consistent with the result in population, P-del construct containing rs10659396 del allele drove significantly higher luciferase expression than P-ins construct and the empty pGL3-promoter plasmid (both *P* < 0.001). About 2-fold higher luciferase expression was observed for P-del construct than P-ins construct, with the values (mean ± SE) being 1.63 ± 0.11 versus 0.80 ± 0.08. Significant disparities were not found between P-ins construct and the empty pGL3-promoter plasmid (*P* = 0.06).

**Fig. 3:**
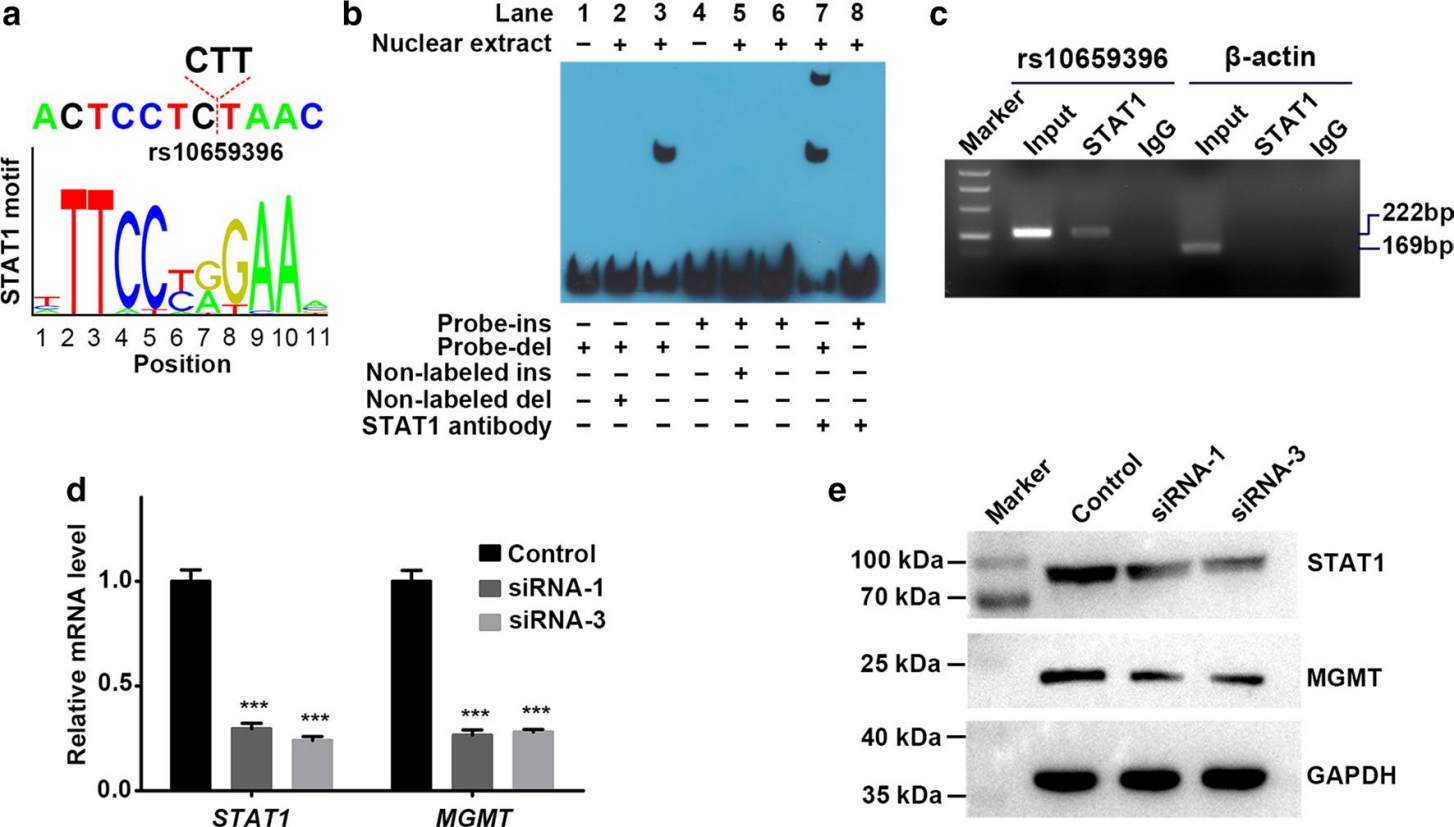
**a** rs10659396 resides within STAT1 DNA-binding motif. **b** EMSA with biotin-labeled oligonucleotides containing the rs10659396 ins allele (Probe-ins) or del allele (Probe-del) and nuclear extracts from U251 cells. Lanes 1 and 4 show mobilities of the labeled oligonucleotides without nuclear extracts; lanes 2 and 5 show mobilities of the labeled oligonucleotides with nuclear extracts in the presence of Non-labeled del or Non-labeled ins competitors; lanes 3 and 6 show mobilities of the labeled oligonucleotides with nuclear extracts in the absence of competitor; lanes 7 and 8 show mobilities of the labeled oligonucleotides with nuclear extracts in the presence of STAT1 antibody. **c** ChIP assays using U251 cells with rs10659396 del/del genotype. Representative gel shows the PCR verification results from input, STAT1 ChIPed DNA, and nonspecific IgG ChIPed DNA. **d** Knockdown of *STAT1* by RNAi diminishes the mRNA levels of *MGMT*. Columns indicate means; Bars represent SE. ^***^P < 0.001. **e** Depletion of STAT1 by RNAi decreases in the protein levels of MGMT

In silico analysis revealed that rs10659396 is within the binding motif of a transcription factor STAT1 (Fig. 3a). EMSA was then conducted to distinguish rs10659396 allelic difference in binding affinity to nuclear proteins. As shown in Fig. 3b, a DNA-protein complex formed with the rs10659396 del probe was detected (Lane 3). Competition assays showed that 200-fold molar excess of non-labeled rs10659396 del probe eliminated the DNA-protein interaction completely (Lane 2). To verify whether STAT1 binds to rs10659396 region in vitro, supershift assays with STAT1 antibody were performed. The addition of STAT1 antibody generated a supershifted complex formed with the rs10659396 del probe (Lane 7). However, under the same experimental conditions, neither shifted nor supershifted complex formed with rs10659396 ins probe was observed (Lane 6, 8).

ChIP assays were performed in U251 cells with rs10659396 del/del genotype to further verify whether STAT1 binds to rs10659396 locus in vivo. We found that rs10659396 locus was precipitated specifically with STAT1 antibody but not nonspecific IgG antibody, demonstrating the existence of STAT1 binding to rs10659396 del allele in vivo (Fig. 3c). On the other hand, no obvious binding was revealed between STAT1 and nonspecific region of β-actin promoter, ruling out nonspecific antibody-DNA interaction.

To evaluate the effect of STAT1 on *MGMT* expression, we designed three siRNAs to knockdown *STAT1* expression. The interference efficiency of siRNAs was assessed by RT-qPCR and Western blotting. The siRNA-1 and siRNA-3 were chosen for further analysis because of higher interference efficiency (Additional file 6). After transfecting siRNA-1 and siRNA-3, the relative mRNA levels of *STAT1* were significantly decreased with the values (mean ± SE) being 0.30 ± 0.03 and 0.24 ± 0.02 respectively (both *P* < 0.001, Fig. 3d). Meanwhile, the relative mRNA levels of *MGMT* were also significantly downregulated with the values being 0.27 ± 0.02 and 0.28 ± 0.01 respectively (both *P* < 0.001, Fig. 3d). Consistently, Western blotting also showed that the protein levels of *MGMT* were obviously downregulated upon knockdown of *STAT1* (Fig. 3e). These results suggested that *MGMT* expression were regulated by transcription factor STAT1 in cell line.

**Fig. 4:**
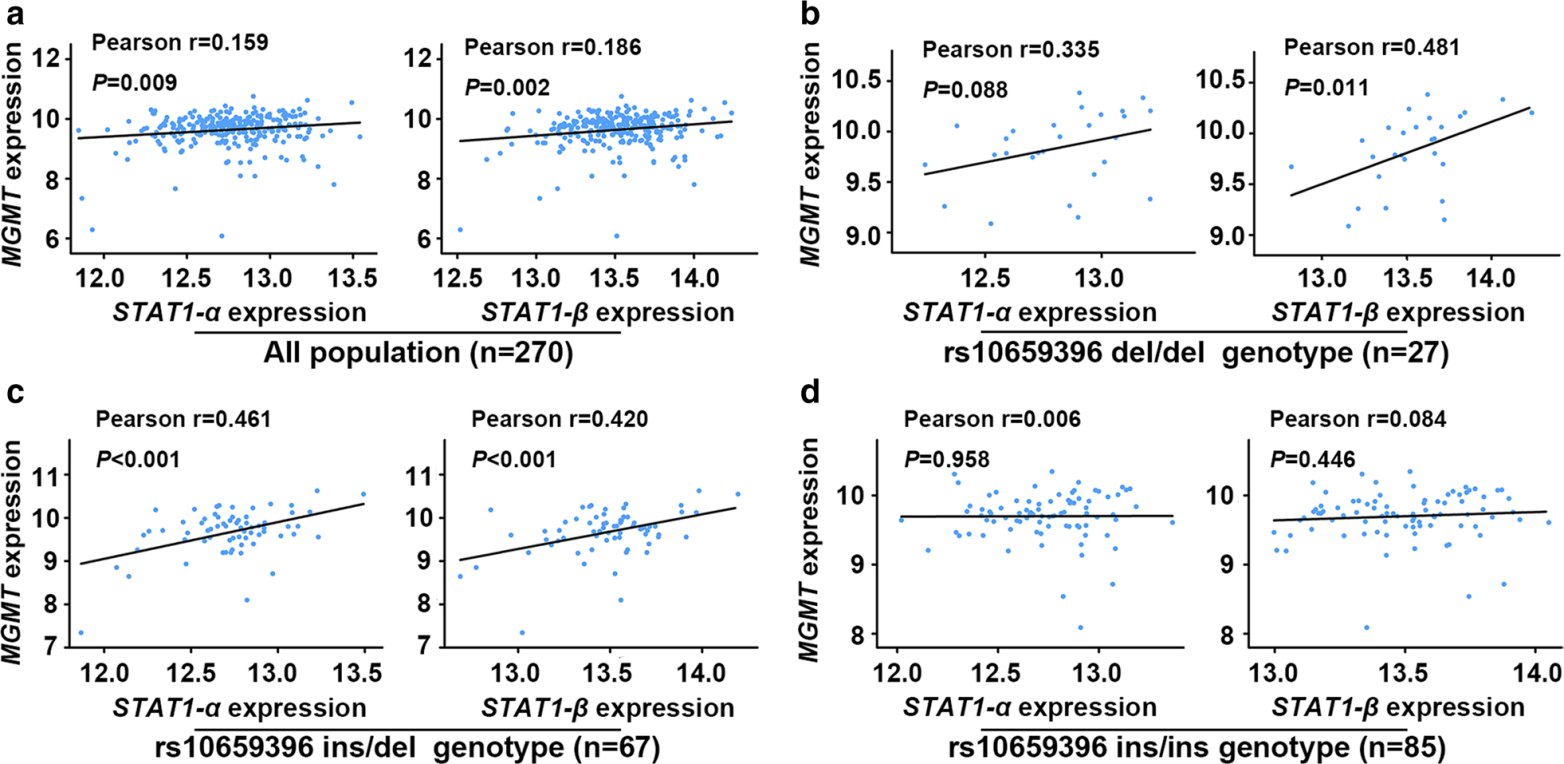
Scatterplots showing an expression correlation between *STAT1* and *MGMT* in different subjects. **a** All population (n = 270). **b** Subjects with rs10659396 del/del genotype (n = 27). **c** Subjects with rs10659396 ins/del genotype (n = 67). **d** Subjects with rs10659396 ins/ins genotype (n = 85)

Then, we examined whether the expression of *STAT1* and *MGMT* are positively correlated in a cohort of 270 unrelated HapMap individuals. As shown in Fig. 4a, in all population (n = 270), positive correlations were observed between the expression of *MGMT* and *STAT1* isoforms, with Pearson r values being 0.159 (*STAT1-α*, *P* = 0.009) and 0.186 (*STAT1-β*, *P* = 0.002) respectively. Interestingly, in subjects with genotype data of rs10659396 (n = 179), we found that the correlations between the expression of *MGMT* and *STAT1* differed across different populations due to rs10659396 genotypes. In subjects with rs10659396 del/del genotype (n = 27), a significant positive correlation was found between the expression of *MGMT* and *STAT1-β* (Pearson r = 0.481; *P* = 0.011) (Fig. 4b). While the positive correlation between the expression of *MGMT* and *STAT1-α* in subjects with rs10659396 del/del genotype only showed a trend towards significance (Pearson r = 0.335; *P* = 0.088) (Fig. 4b), which might be due to the small sample size within this stratum. In subjects with rs10659396 ins/del genotype (n = 67), both *STAT1-α* and *STAT1-β* had significant positive correlations with *MGMT*, with Pearson r values being 0.461 and 0.420 respectively (both *P* < 0.001, Fig. 4c). Consistent with our previous results, no significant correlation was observed between the expression of *MGMT* and *STAT1* isoforms in subjects with rs10659396 ins/ins genotype (n = 85, Fig. 4d).

## Discussion

In the present study, we conducted a case–control study consisting of 402 glioma patients and 654 controls to investigate the associations of the eQTLs of *MGMT* with glioma susceptibility. We demonstrated that an eQTL of *MGMT* rs11016798 was significantly associated with glioma susceptibility. An insertion variant rs10659396, which is in perfect linkage disequilibrium with rs11016798, was identified as the causative variant. The rs10659396 ins allele was found to downregulate *MGMT* expression by disrupting a STAT1 binding site.

DNA damage repair is a fundamental mechanism to ensure the accuracy of genetic information transmission. The deficiency in the DNA repair pathway results in accumulation of deleterious mutations, and then leads to cancer [26]. As the pivotal repair gene for DNA alkylation damage, *MGMT* inactivation has been reported to be a common event in several kinds of cancer and associated with increased frequency of tumor-associated genes mutations [6, 27, 28]. On the other hand, the reduced cancer risk due to *MGMT* overexpression also reveals its tumor suppress activity [7, 29]. Consistently, our data demonstrated that rs11016798 TT genotype associated with higher *MGMT* expression decreased the risk of developing glioma. Although the other 3 tag-eQTLs were not found to be significantly associated with glioma susceptibility, one of them rs557311 approached the borderline of significance. More to the point, a consistent trend was observed in all 4 tag-eQTLs that subjects carrying the genotypes associated with higher *MGMT* expression were more likely to have decreased risk of developing glioma. Therefore, it is worthy to carry out further studies to evaluate the exact role of the other 3 tag-eQTLs in glioma carcinogenesis.

*IDH* mutation has been known as an early event in tumuorigenesis of many gliomas. Glioma with *IDH* mutation is possessed of a particular mechanism for oncogenic progression and a favorable prognosis [30]. In the present study, the association between rs11016798 and glioma was found to be more pronounced in IDH wildtype glioma. It also indicates the distinct biology between IDH wildtype glioma and IDH mutant glioma. Moreover, *IDH* mutation has been found to be prevalent in WHO grade II and III glioma patients, whereas it is rare in the primary WHO grade IV glioblastoma observed in older patients [31–33]. Consistently, more pronounced decreased risk of glioma was observed in older subjects and WHO grade IV glioma in the present study. Nevertheless, it should be noted that a consistent trend was found in all subgroups that subjects with rs11016798 TT genotype were more likely to have decreased risk of glioma. Because of the relatively small sample size within a given subgroup, further studies are warranted to confirm whether rs11016798 exerts a different effect on glioma carcinogenesis due to different IDH status. The functional variant rs10659396 located in the upstream of *MGMT* was recognized as the causative variant in the present study. Both in cell line and in population, consistent results were found that rs10659396 ins allele drove significantly lower gene expression. A set of biochemical assays revealed that the reduced gene expression driven by rs10659396 ins allele may resulted from the disruption of a binding site for STAT1, a transcriptional factor that has been shown to promote specific gene expression [34]. STAT1 has been demonstrated to perform important roles in several critical cellular processes, such as cell death, growth, and differentiation [34]. While there is still controversy concerning the actual role of STAT1 in carcinogenesis, the majority of studies recognize STAT1 as a tumor suppressor. For instance, activation of STAT1 was reported to suppress the proliferation of glioma cells [35]. A more convincing evidence is that STAT1 knockout mice are highly susceptible to some tumours [36, 37]. In the present study, we found that STAT1 bound to rs10659396 del allele and upregulated *MGMT* expression. Logically, rs10659396 del allele associated with higher *MGMT* expression was found to decrease the risk of developing glioma. These molecular epidemiological results provide logical evidence to support the contention that STAT1 may mediate its tumor suppressor function in glioma by regulating *MGMT* expression.

## Conclusions

An insertion variant of *MGMT* rs10659396 was identified as a novel susceptibility locus for glioma. Significantly decreased risk of glioma was observed in males, older subjects, WHO grade IV glioma, and IDH wildtype glioma subgroups. The three-nucleotide insertion disrupts a STAT1 binding site and downregulates *MGMT* expression. After all, our findings bring the new insight that STAT1 may repress glioma carcinogenesis by modulating *MGMT* expression.

## Supplementary Information

**Additional file 1:Table S1. **Selected characteristics of glioma patients and controls.

**Additional file 2:Table S2. **Oligonucleotides for EMSA, ChIP-PCR, RNAi, and RT-qPCR.

**Additional file 3: Figure S1.** Associations of the 4 tag-eQTLs with *MGMT* expression levels. This figure was modified from BrainSeq project (http://eqtl.brainseq.org/). Inside lines of boxes indicate medians; Upper and lower limits of boxes represent the 75th and 25th percentiles respectively; Vertical bars indicate maximum and minimum values; Points represent outlier values.

**Additional file 4:Figure S2. **In silico analysis of rs11016798.

**Additional file 5:Figure S3. **In silico analysis of rs10659396.

**Additional file 6:Figure S4.** Interference efficiency of the candidate STAT1 silencing RNA oligonucleotides. (a) The relative mRNA levels of STAT1 after transfecting siRNA-1, siRNA-2, and siRNA-3. Columns indicate means; Bars represent SE. (b)The protein levels of STAT1 after transfecting siRNA-1, siRNA-2, and siRNA-3.

## Data Availability

The datasets used and/or analyzed during the current study are available from the corresponding author on reasonable request.

## Abbreviations

MGMT: *O*^6^-methylguanine-DNA methyltransferase
eQTLs: Expression quantitative trait loci
CHS: Southern Han Chinese
MAF: Minor allelic frequency
LD: Linkage disequilibrium
EMSA: Electrophoretic mobility-shift assays
ChIP: Chromatin immunoprecipitation
siRNA: Silencing RNA
GEO: Gene Expression Omnibus
OR: Odds ratios
CI: Confidence interval
IDH: Isocitrate dehydrogenase
