# Supplementary material for: An insertion variant of MGMT disrupts a STAT1 binding site and confers susceptibility to glioma
Source: Cancer Cell Int. 2021 Sep 20;21:506. doi: 10.1186/s12935-021-02211-4 (PMC8454171; doi:10.1186/s12935-021-02211-4)
Supplement: Supplementary file 2 — Additional file 2:Table S2. Oligonucleotides for EMSA, ChIP-PCR, RNAi, and RT-qPCR. [file 12935_2021_2211_MOESM2_ESM.docx]

**Table S2.** Oligonucleotides for EMSA, ChIP-PCR, RNAi, and RT-qPCR

| Experiment | Designation | Sequence (5’-3’) |
| --- | --- | --- |
| EMSA | Probe-del | biotin-GTCACTCCTCTAACCCACTC |
|  | Probe-ins | biotin-GTCACTCCTCCTTTAACCCACTC |
|  | Non-labeled del | GTCACTCCTCTAACCCACTC |
|  | Non-labeled ins | GTCACTCCTCCTTTAACCCACTC |
| ChIP | rs10659396-F | TCAGTCACTGTCCACTGCAC |
|  | rs10659396-R | CGGACCACCTCATTGATTCATTC |
|  | β-actin-F | tgacaaggacagggtcttcc |
|  | β-actin-R | caccgtccgttgtatgtctg |
| RNAi | siRNA-1 | GCGUAAUCUUCAGGAUAAU UU |
|  | siRNA-2 | UCCAGAUGUCUAUGAUCAU UU |
|  | siRNA-3 | GCAGGUUCACCAGCUUUAU UU |
|  | siRNA-Control | UUCUCCGAACGUGUCACGU dTdT |
| RT-qPCR | STAT1-F | ggcacgcacacaaaagtgat |
|  | STAT1-R | ttggagatcaccacaacggg |
|  | MGMT-F | ACCGTTTGCGACTTGGTACT |
|  | MGMT-R | GGGCTGGTGGAAATAGGCAT |
|  | GAPDH-F | AACGGATTTGGTCGTATTGGG |
|  | GAPDH-R | CCTGGAAGATGGTGATGGGAT |
|  |  |  |
