# Supplementary figures and images for: An insertion variant of MGMT disrupts a STAT1 binding site and confers susceptibility to glioma
Source: Cancer Cell Int. 2021 Sep 20;21:506. doi: 10.1186/s12935-021-02211-4 (PMC8454171; doi:10.1186/s12935-021-02211-4)

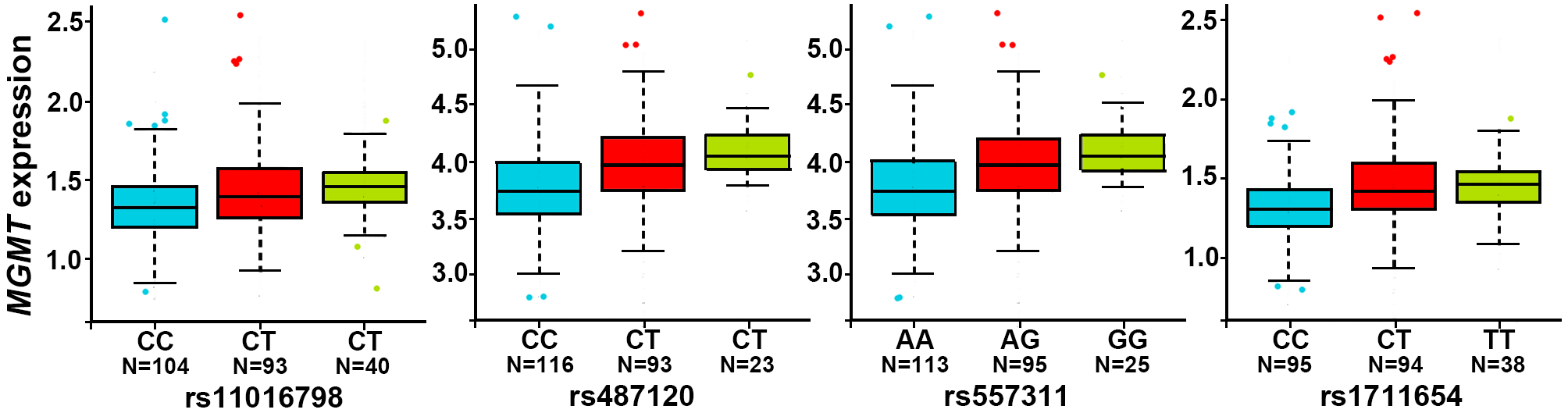

Supplement: Supplementary file 3 — Additional file 3: Figure S1. Associations of the 4 tag-eQTLs with MGMT expression levels. This figure was modified from BrainSeq project (http://eqtl.brainseq.org/). Inside lines of boxes indicate medians; Upper and lower limits of boxes represent the 75th and 25th percentiles respectively; Vertical bars indicate maximum and minimum values; Points represent outlier values. [file 12935_2021_2211_MOESM3_ESM.tif]

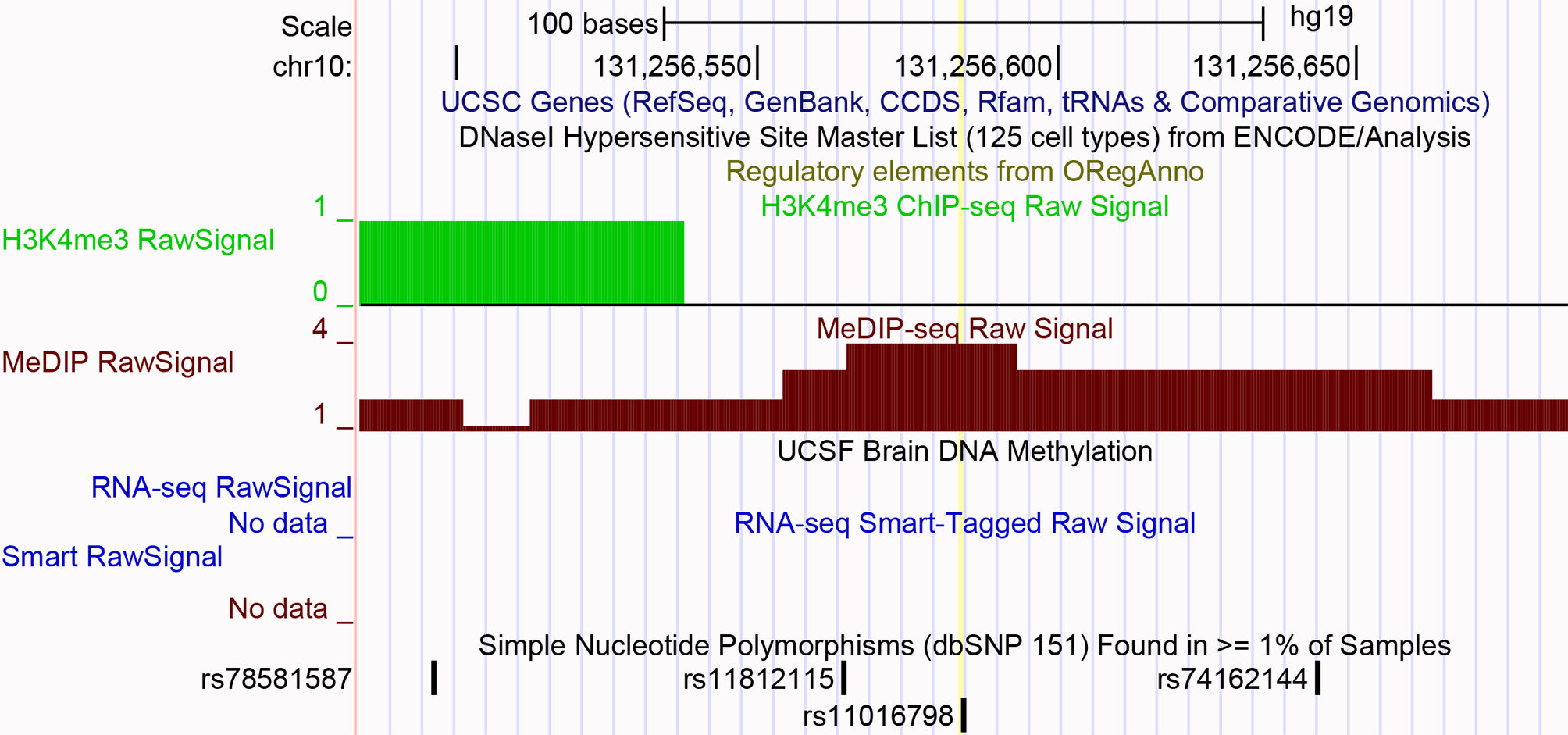

Supplement: Supplementary file 4 — Additional file 4:Figure S2. In silico analysis of rs11016798. [file 12935_2021_2211_MOESM4_ESM.tif]

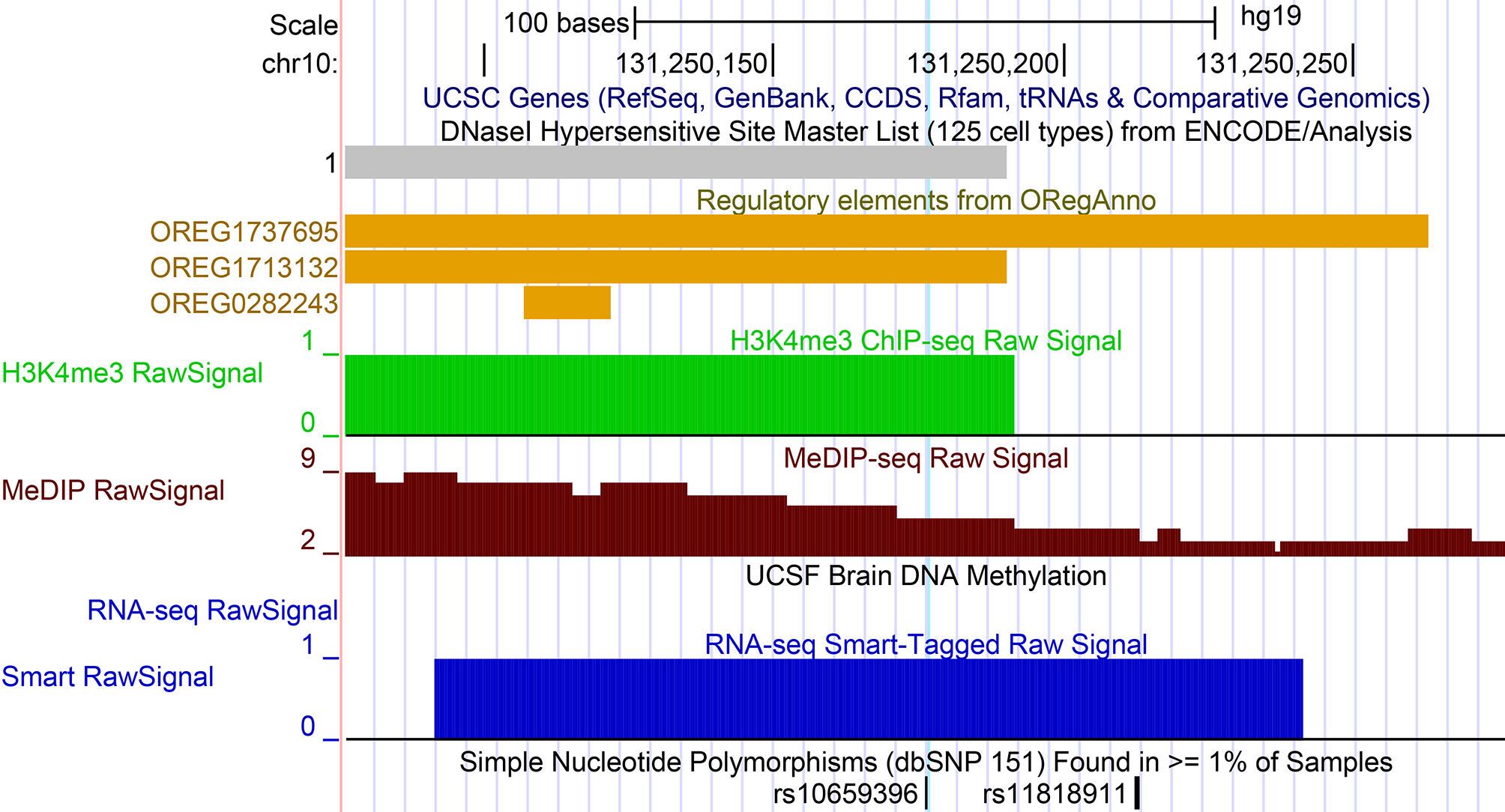

Supplement: Supplementary file 5 — Additional file 5:Figure S3. In silico analysis of rs10659396. [file 12935_2021_2211_MOESM5_ESM.tif]

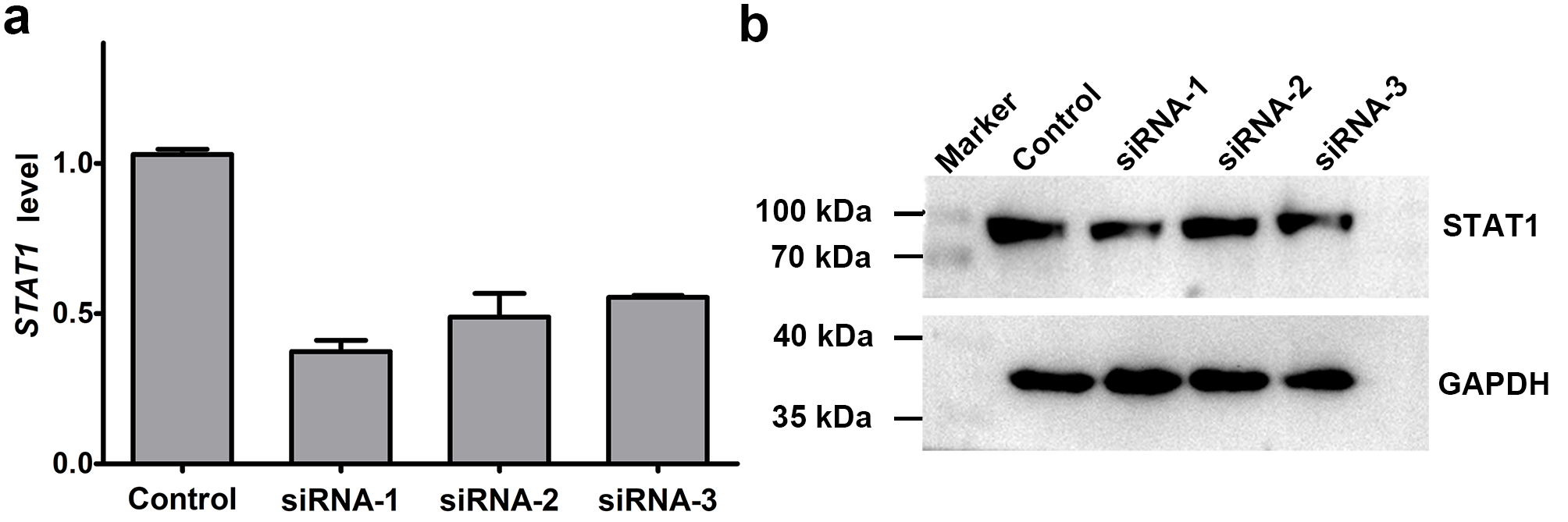

Supplement: Supplementary file 6 — Additional file 6:Figure S4. Interference efficiency of the candidate STAT1 silencing RNA oligonucleotides. (a) The relative mRNA levels of STAT1 after transfecting siRNA-1, siRNA-2, and siRNA-3. Columns indicate means; Bars represent SE. (b)The protein levels of STAT1 after transfecting siRNA-1, siRNA-2, and siRNA-3. [file 12935_2021_2211_MOESM6_ESM.tif]
